# Differences between CS-DAVF and TCCF——to reveal and redefine CS-DAVF

**DOI:** 10.1186/s41016-018-0121-z

**Published:** 2018-06-11

**Authors:** Li Pan, Jian Peng Wen, Lian Ting Ma

**Affiliations:** Department of Neurosurgery, Wuhan General hospital of Guangzhou Military, Wuhan, 430061 Hubei Province China

**Keywords:** Traumatic carotid cavernous fistula, Cavernous dural arteriovenous fistula, Differentiation

## Abstract

In the past, the cavernous dural arteriovenous fistula was categorized as spontaneous cavernous carotid fistula [1] due to the lack of knowledge and limitation of imaging equipment. In the recent time, with the accumulation of knowledge of DAVF’s etiology, mechanism, physiology, clinical symptoms and imaging data, the diagnostic methods and treatment have achieved novel understandings and progresses. In fact, it’s a specific type of dural arteriovenous fistula---- cavernous dural arteriovenous fistula. The purpose of this paper is to tell the difference between cavernous dural arteriovenous fistula and traumatic carotid cavernous fistula, and to redefine cavernous dural arteriovenous fistula from the aspects of etiology, mechanism, pathology, clinical symptoms, DSA characteristic and therapy. DAVF is an independent disease. The Cavernous dural arteriovenous fistula can not be classified as spontaneous CCF any longer, but a specific type of DAVF--- cavernous DAVF.

## Etiology

TCCF is mainly caused by trauma and skull base fracture.

CS-DAVF is caused by cerebral venous sinus thrombosis, venous sinus hypertension, inflammation, trauma or endocrine diseases, etc.

## Mechanism

TCCF: Skull base fracture injures the internal carotid artery and its branches, which gives rise to the abnormal communication between ICA and cavernous sinus.

CS-DAVF: Multiple reasons lead to the communication between dural neoformative multi-branch arteriole and cavernous sinus through the dural microfistulae.

## Pathology

TCCF: The feeding artery is cavernous segment of internal carotid artery or its branch-vessels; In few cases, the feeding artery is the external carotid artery; Normally single fistulous orifice; The draining veins are superior ophthalmic vein, superior petrosal sinus, inferior petrosal sinus,basal veins, cortex veins (labbe, Tralend veins) or mixed-type draining veins (two, three or four veins of the four draining veins mentioned above).

**Fig. 1:**
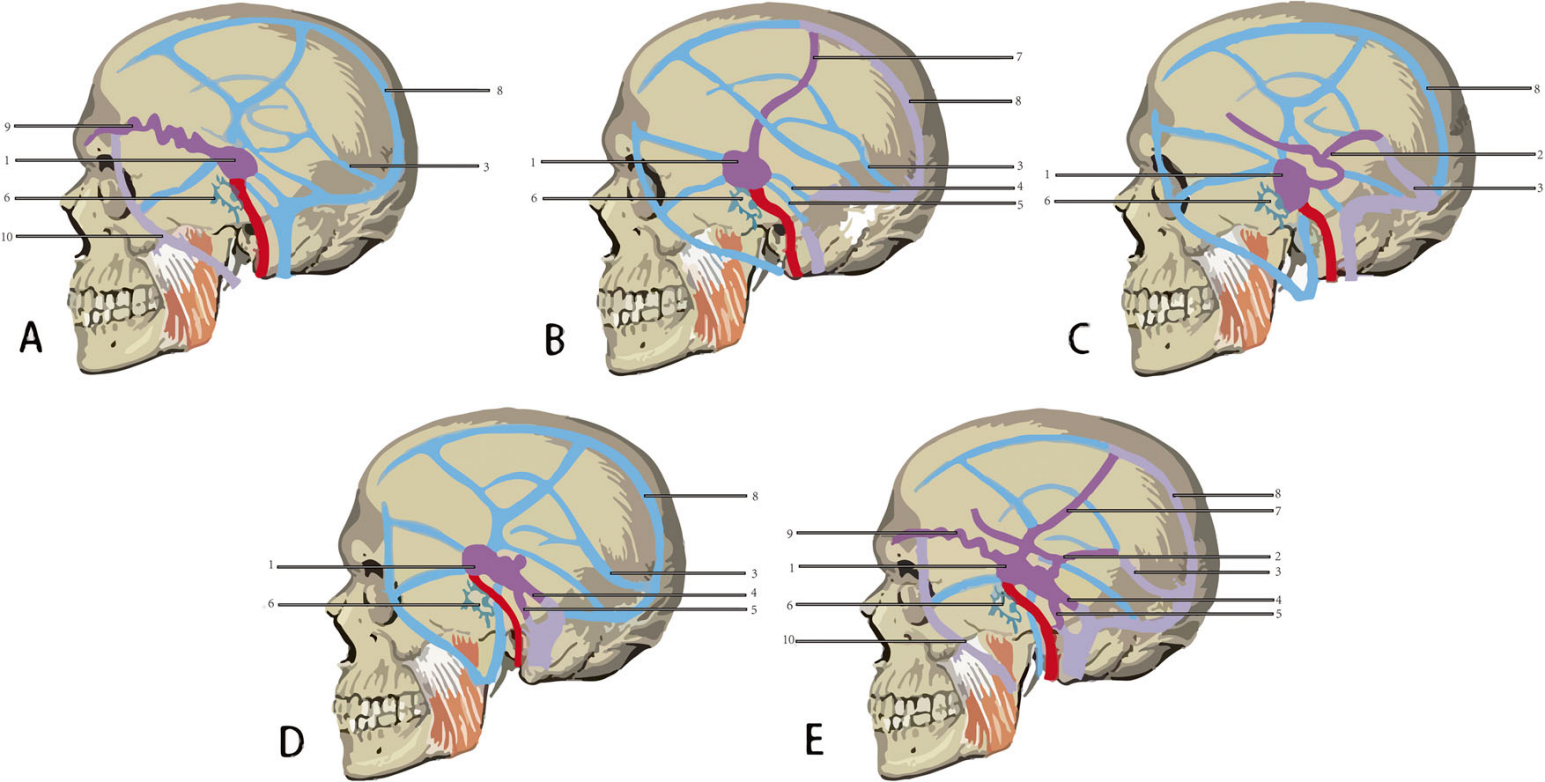
The anastomosis of cavernous region [3] and illustration of ICA-cavernous fistula. **a** Branches of cavernous segment of Internal carotid artery (1. meningopituitary trunk arter,including: ①inferior hypophyseal artery, ②dorsal meningeal artery, ③tentorial artery; 2. inferior cavernous sinus artery; 3. capsular arteries; 4. Internal carotid artery; 5. Ophthalmic artery;6. pituitary); **b** Cavernous sinus (internal basal surface of skull; including: 1. Superior ophthalmic vein, 2. Anterior intercavernous sinus; 3. pituitary; 4. Cavernous sinus; 5. Posterior intercavernous sinus; 6. Plexus basilaris; 7. Inferior petrosal sinus; 8. Glossopharyngeal nerve; 9. Vagus nerve; 10. accessory; 11. Hypoglossal nerve; 12. tentoriumcerebelli; 13. Sphenoparietal sinus; 14. Optic nerve; l5. Internal carotid artery; 16. Posterior clinoid process; 17. Oculomotor nerve; 18. Trochlear nerve; 19. Trigeminal nerve; 20. Abducent nerve; 21. Superior petrosal sinus; 22. Facial nerve 、acoustic nerve; 23. sigmoid sinus; 24. great cerebral vein; 25. sinus rectus); **c** Cavernous sinus (coronal section; 1. pituitary; 2. Internal carotid artery; 3. Aducent nerve; 4. Sphenoid sinus; 5. Sphenoid bone; 6. Oculomotor nerv; 7. Trochlear nerve; 8. Ophthalmic branch of trigeminal nerve); **d** Topography of cavernous sinus (relationship between ICA and cerebral nerve; 1. Superior wall of cavernous sinus; 2. Oculomotor nerve; 3. Trochlear nerve; 4. Aducent nerve; 5. ophthalmic branch of trigeminal nerve; 6. Trigeminal semilunar ganglion; 7. Mandibular branch of trigeminal nerve; 8. Internal carotid artery; 9. Ophthalmic artery; 10. optic nerve; 11. Superior optical fissure; 12. Maxillary branch of tigeminal nerve; **e** Carotid-cavernous fistula (1. ICA;2. Fistulous orifice;3. Cavernous sinus)

**Fig. 2:**
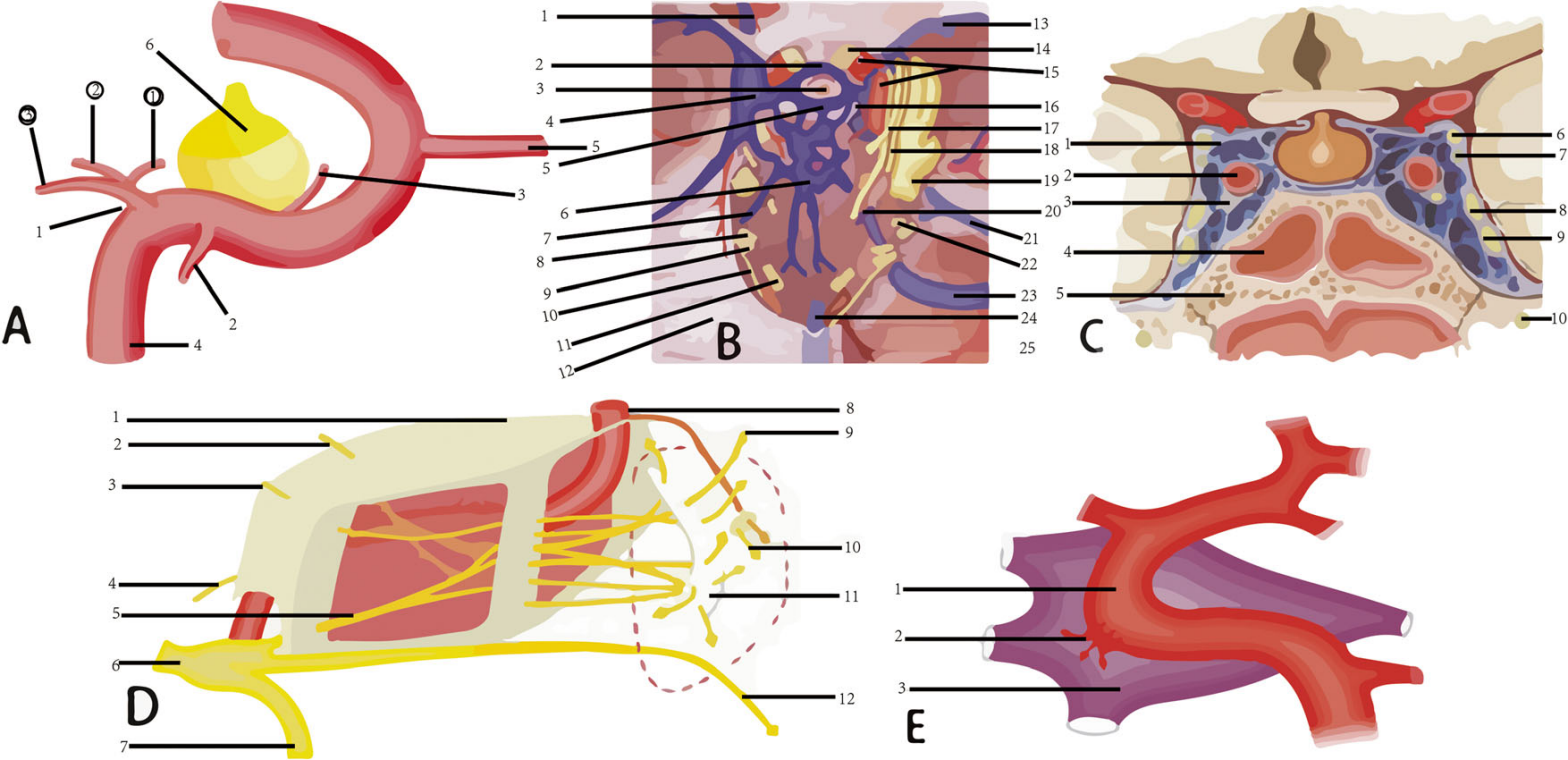
Five types of venous draining of cavernous fistula. **a** Arterial blood is drained to facial vein from cavernous sinus through superior ophthalmic vein and angular vein; **b** arterial blood is drained to superior sagittal sinus from cavernous sinus through trolard frontal parietal anastomotic vein. **c** arterial blood is drained to basilar vein from cavernous sinus through an anastomotic vein, and together with Vein of Galen , drained to straight sinus. **d** arterial blood is drained to internal carotid vein from cavernous sinus through superior petrosal vein, inferior petrosal vein, basilar vein and plexus pterygoideusta. **e** A mixed type: Two kinds of draining together of the four mentioned above. 1. cavernous sinus; 2. basilar vein; 3. straight sinus; 4. superior petrosal sinus; 5. inferior petrosal sinus; 6. plexus pterygoideusta’7. Trolard frontal parietal anastomotic vein; 8.superior sagittal sinus; 9. superior ophthalmic vein; 10. facial vein

CS-DAVF: In most cases, the feeding arteries are the ascending pharyngeal artery, sphenopalatine artery and the middle meningeal artery of external carotid artery. There are also the tiny branch arteries from the meningopituitary trunk feeding the CS-DAVF; Normally multiple microporous fistulous orifices. The draining veins are mainly superior ophthalmic vein, superior petrosal sinus and inferior petrosal sinus. Basal vein and cortex veins are not frequently seen. The contralateral internal (external) carotid artery are often feeders when bilateral cavernous sinuses are involved by the lesion; Stealing symptom is not manifest (Figs. 1 and 2).

## Clinical symptoms

TCCF: intracranial bruit;exophthalmos; obvious hyperemia and edema of bulbar conjuctive and eyelid; elevation of intraorbital pressure; restriction of occular motility; sometimes intracranial hemorrhage or steal syndrome.

CS-DAVF: mild and rare intracranial bruit; rare hyperemia and edema of bulbar conjuctive and eyelid; rare intracranial hemorrhage or steal syndrome; easyily misdiagnosed as conjuctivitis.

## DSA characteristic

**Fig. 3:**
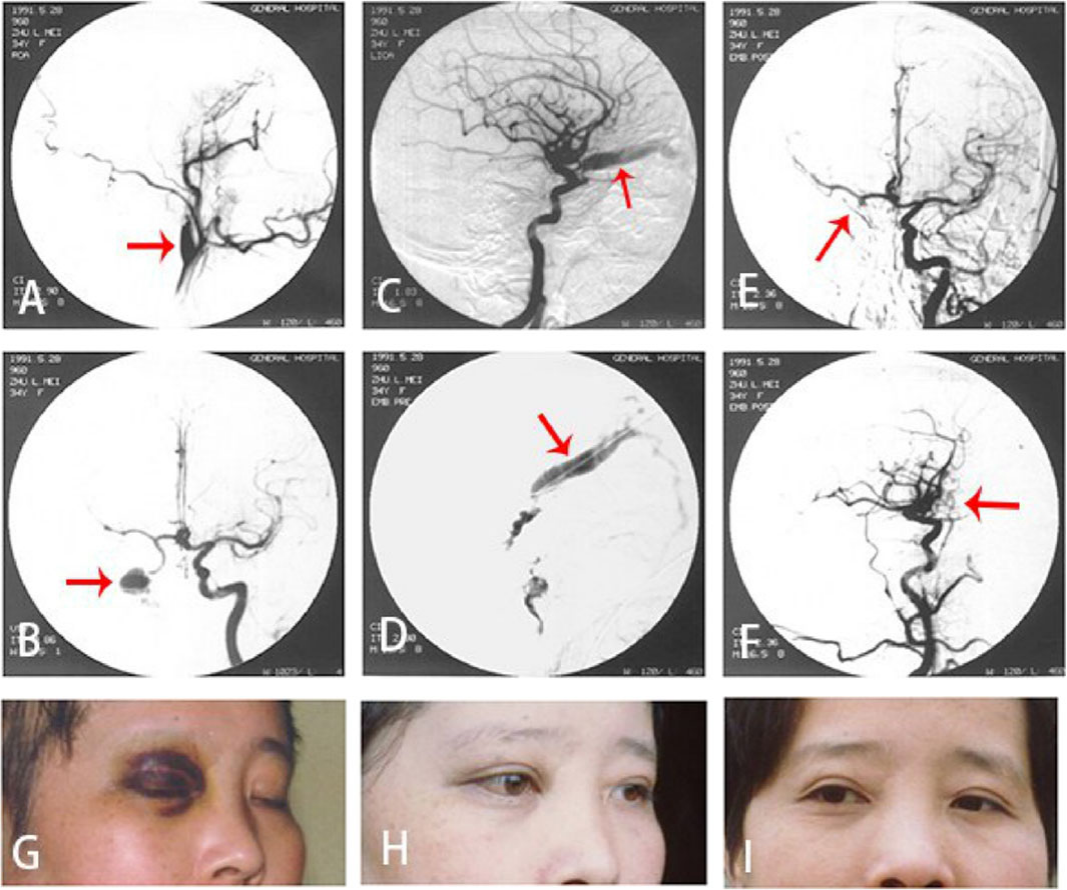
The DSA and clinical symptoms before and after the embolization of right ICA cavernous sinus fistula. **a** Right carotid cavernous sinus; Arrow shows misembolization of internal carotid artery in other hospitals. **b** Arrow shows angiography of left internal carotid artery: right cavernous sinus fistula is opacified through anterior communicating artery. **c** Angiography of left internal carotid artery, right cavernous sinus is opacified through ACA. Arrow shows dilated draining of superior ophthalmic vein. **d** Directly puncture to the fistulous orifice through superior ophthalmic vein and inject the NBCA to occlude the fistula. Arrow shows the catheter. **e** Angiography of left internal carotid artery after embolization. The right cavernous sinus fistula is not opacified. Arrow shows the fistulous orifice is occluded. **f** Angiography of left internal carotid artery after embolization. The right cavernous sinus fistula is not opacified. Arrow shows superior ophthalmic vein is vanished. **g** The ophthalmic symptoms before embolization. **h** The ophthalmic symptoms are gone 2 weeks after embolization. **i**. Follow-up 5 years after embolization

**Fig. 4:**
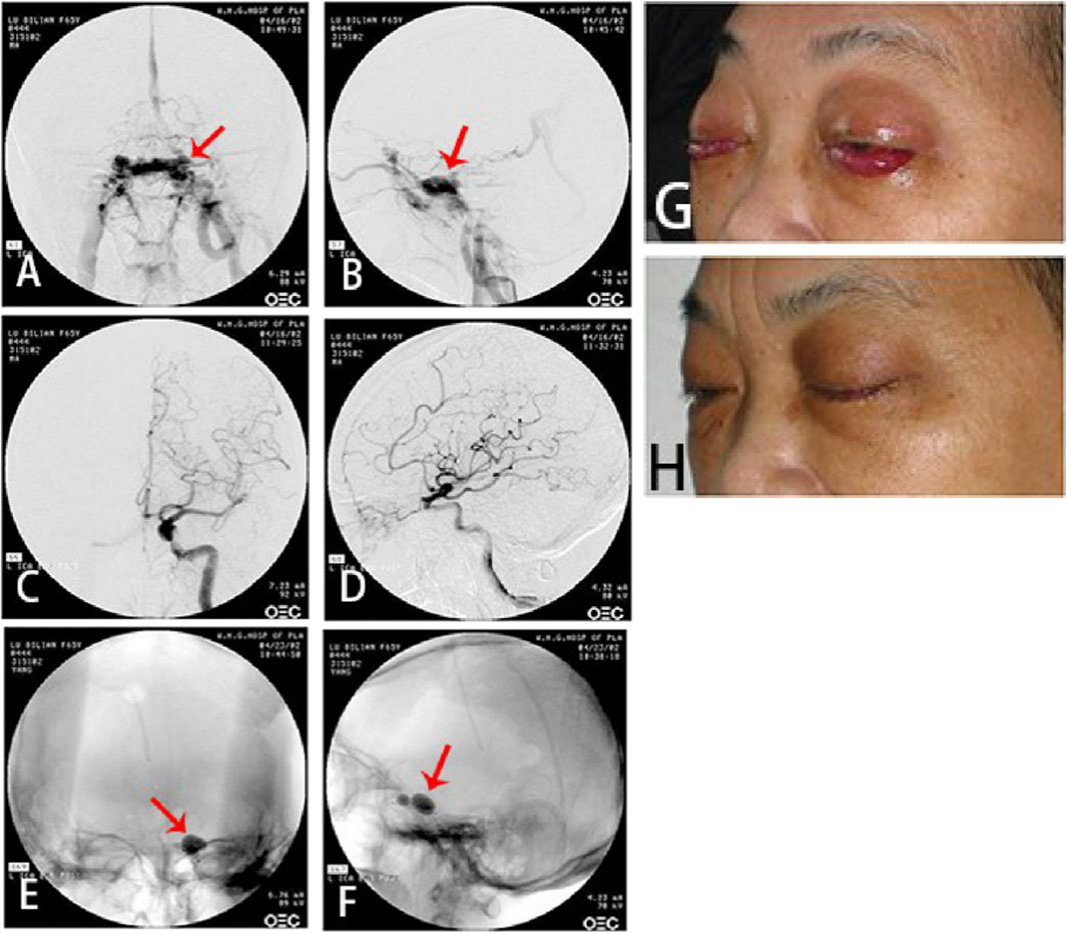
The DSA and clinical symptoms before and after the embolization of left ICA cavernous sinus fistula. **a** Left internal carotid artery in orthophoria view. Arrow shows the left internal carotid cavernous sinus fistula opacify the contralateral cavernous sinus through intervcavernous sinus. **b** Arrow shows angiography of left internal carotid artery in lateral view. **c** The DSA in orthophoria view shows the fistulous orifice vanish and the internal carotid artery is unobstructed after detachable balloon embolization. **d** The DSA in lateral view shows the fistulous orifice vanish and the internal carotid artery is unobstructed after detachable balloon embolization. **e** X ray film in orthophoria view. Arrow shows the balloon. **f** X ray film in lateral view 1 week after embolization. Arrow shows the balloon. **g** Eye’s edema and hyperemia before embolization. **h** 1 week after embolization

**Fig. 5:**
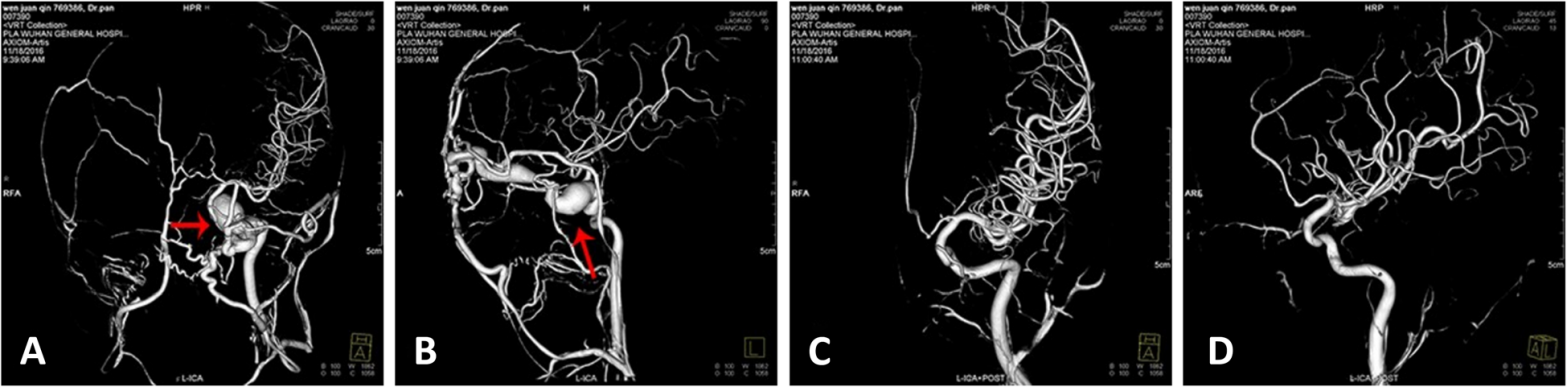
TCCF. **a** The static DSA image in orthophoria view before treatment. Red arrow shows the fistulous orifice; **b** The static DSA image in lateral view before treatment. Red arrow shows the fistulous orifice. **c** The static DSA image in orthophoria view after treatment. **d** The static DSA image in lateral view before treatment

The feeding artery of TCCF is the cavernous segment of internal carotid artery, mostly the horizontal segment. Single fistulous orifice, mostly on the side near skull base. The draining veins are mainly superior ophthalmic vein, superior petrosal sinus, inferior petrosal sinus, sometimes cortex veins and basilar vein (Figs. 3a-i, 4a-h, 5a-d).

**Fig. 6:**
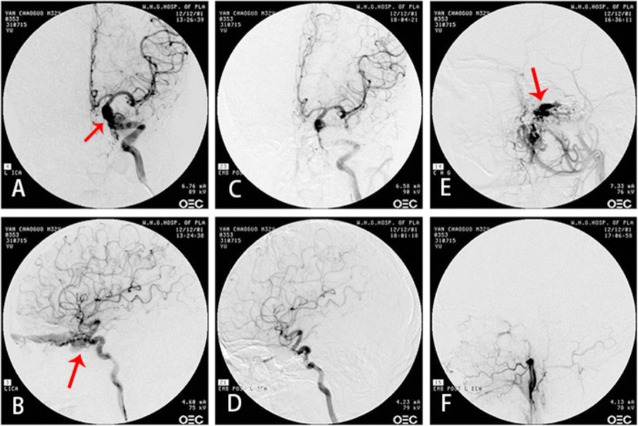
The DSA before and after CS-DAVF embolization. **a**, **b** The DSA of left internal carotid artery in orthophoria and lateral view before embolization. Arrow shows the fistulous orifice. **c**, **d** The DSA of left internal carotid artery in orthophoria and lateral view after embolization. **e** The DSA of left external carotid artery in orthophoria and lateral view before embolization. Arrow shows the fistulous orifice. **f** The DSA of left external carotid artery in orthophoria and lateral view after embolization

**Fig. 7:**
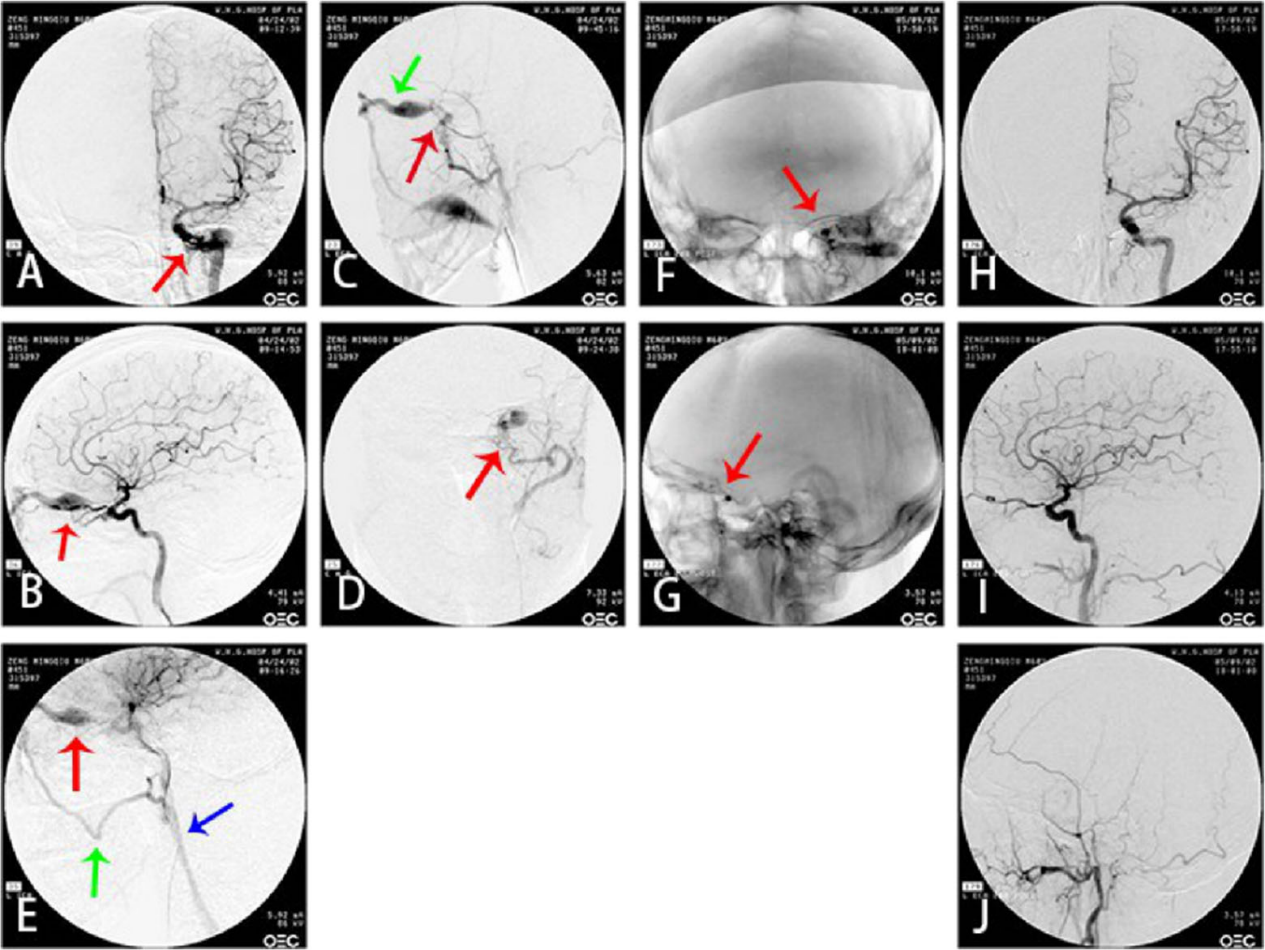
CS-DAVF. **a**, **b** The DSA of left internal carotid artery in orthophoria and lateral view. The red arrow shows the fistulous orifice; **c**, **d** The DSA of left external carotid artery in orthophoria and lateral view. Red arrow:The fistulous orifice. Green arrow: superior ophthalmic vein. **e** DSA shows the blood draining route: Superior ophthalmic vein(red arrow)→ superior facial vein(green arrow)→ carotid vein(blue arrow). **f**, **g** The DSA in orthophoria and lateral view of embolization through superior ophthalmic approach. Arrow sho9ws the coils between cavernous sinus and superior ophthalmic vein; **h**, **i** The DSA of left internal carotid artery in orthophoria and lateral view after embolization. **j** Left external carotid artery after embolization

**Fig. 8:**
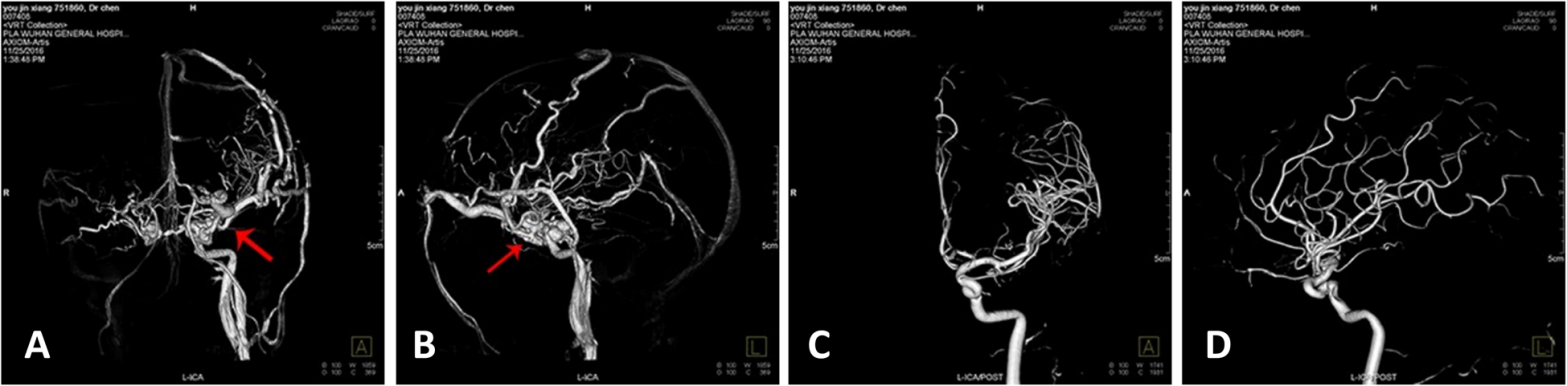
CS-DAVF. **a** The static DSA image in orthophoria view before treatment. Red arrow shows the fistulous orifice; **b** The static DSA image in lateral view before treatment. Red arrow shows the fistulous orifice. **c** The static DSA image in orthophoria view after treatment. **d** The static DSA image in lateral view before treatment

CS-DAVF: The feeding arteries are the branches of external carotid artery. In some few cases, the branches of meningopituitary trunk feed the contralateral synonymous vessels. The fistulous orifices on the dural mater are multi-microporous, which are naked-eye invisible. The draining veins are mainly superior ophthalmic vein, superior petrosal vein and inferior petrosal vein, which can communicates to the contralateral vessels through intercavernous sinus (Figs. 6a-f, 7a-j, 8a-d).

## Therapy

TCCF: Endovascular embolization is the prior therapy and internal carotid artery is the prior approach. But when internal carotid artery approach can not be performed, superior ophthalmic vein, superior petrosal vein and inferior petrosal vein are also optimal approaches. Detachable ballon is the prior embolic material, but in some special cases, coils and onyx glue are also available. Surgical therapy is barely considered. Our hospital has treated TCCF in a total of more than 900 cases. All patients are completely cured. No disability or death is observed.

CS-DAVF:Endovascular treament is the first choice for cavernous DAVF [2]. We can choose arterial approach, venous approach or combined approach. Coils plus onyx glue, silk line segments or grubran glue can be chosen as embolic materials. As there are multiple feeding arteries and fistulous orifices, the remaining fistulous orifices can be cured by pressing the CCA postoperatively (2–3 months, 2 times a day, keep pressing CCA for 30 min every time).

The treatment of CS-DAVF is rather more difficult than TCCF. However, when choosing the proper treatment, CS-DAVF can be cured and zero death can be reached with extremely low complication rate. Because dangerous anastomasis exists between branches of external carotid artery and ophthalmic artery, vertebrobasilar artery, embolic materials would cause misembolization through dangerous anastomasis when arterial embolizaion is performed. Over-packing with coils would cause ocular muscle paralysis or reflux of liquid embolic agent to artery and their correspondent complications. Our hospital has treated cavernous CS-DAVF in a total of 200 cases since 1900. The longest follow-up is 25 years. No death occurred and only 1 case of right eye blindness was observed.

## Conclusion

Based on the above knowledge, we suggest that the term of spontaneous internal carotid cavernous sinus should not be used. DAVF is an independent disease. Do not confuse CS-DAVF with TCCF and categorize CS-DAVF as cavernous type of DAVF when we write papers or books.

## Abbreviations

CCA: Common carotid artery
CCF: Cavernous carotid fistula
CS-DAVF: Cavernous dural arteriovenous fistula
DAVF: Dural arteriovenous fistula
DSA: Digital subtraction angiography
ICA: Internal carotid artery
TCCF: Traumatic carotid cavernous fistula

## References

[1] Barrow DL, Spector RH, Braun IF (1985). Classification and treatment of spontaneous carotid-cavernous sinus fistulas. J Neurosurg.

[2] Harris FS, Rhoton AL (1976). Anatomy of the cavernous sinus. A microsurgical study. J Neurosurg.

[3] Borden JA, Wu JK, Shucart WA (1995). A proposed classification for spinal and cranial dural arteriovenous fistulous malformations and implications for treatment. J Neurosurg.

